# Approaching 18% efficiency of ternary organic photovoltaics with wide bandgap polymer donor and well compatible Y6 : Y6-1O as acceptor

**DOI:** 10.1093/nsr/nwaa305

**Published:** 2020-12-31

**Authors:** Xiaoling Ma, Anping Zeng, Jinhua Gao, Zhenghao Hu, Chunyu Xu, Jae Hoon Son, Sang Young Jeong, Caixia Zhang, Mengyang Li, Kai Wang, He Yan, Zaifei Ma, Yongsheng Wang, Han Young Woo, Fujun Zhang

**Affiliations:** Key Laboratory of Luminescence and Optical Information, Ministry of Education, Beijing Jiaotong University, Beijing 100044, China; Departmentof Chemistry, The Hong Kong University of Science and Technology, Hong Kong, China; Key Laboratory of Luminescence and Optical Information, Ministry of Education, Beijing Jiaotong University, Beijing 100044, China; Key Laboratory of Luminescence and Optical Information, Ministry of Education, Beijing Jiaotong University, Beijing 100044, China; Key Laboratory of Luminescence and Optical Information, Ministry of Education, Beijing Jiaotong University, Beijing 100044, China; Organic Optoelectronic Materials Laboratory, Department of Chemistry, College of Science, Korea University, Seoul 02841, South Korea; Organic Optoelectronic Materials Laboratory, Department of Chemistry, College of Science, Korea University, Seoul 02841, South Korea; Key Laboratory of Luminescence and Optical Information, Ministry of Education, Beijing Jiaotong University, Beijing 100044, China; Center for Advanced Low-Dimension Materials, State Key Laboratory for Modification of Chemical Fibers and Polymer Materials, College of Materials Science and Engineering, Donghua University, Shanghai 201620, China; Key Laboratory of Luminescence and Optical Information, Ministry of Education, Beijing Jiaotong University, Beijing 100044, China; Departmentof Chemistry, The Hong Kong University of Science and Technology, Hong Kong, China; Institute of Polymer Optoelectronic Materials and Devices, State Key Laboratory of Luminescent Materials and Devices, South China University of Technology, Guangzhou 510640, China; Center for Advanced Low-Dimension Materials, State Key Laboratory for Modification of Chemical Fibers and Polymer Materials, College of Materials Science and Engineering, Donghua University, Shanghai 201620, China; Key Laboratory of Luminescence and Optical Information, Ministry of Education, Beijing Jiaotong University, Beijing 100044, China; Organic Optoelectronic Materials Laboratory, Department of Chemistry, College of Science, Korea University, Seoul 02841, South Korea; Key Laboratory of Luminescence and Optical Information, Ministry of Education, Beijing Jiaotong University, Beijing 100044, China

**Keywords:** organic photovoltaics, power conversion efficiency, ternary strategy, photon harvesting, morphology regulation

## Abstract

A series of ternary organic photovoltaics (OPVs) are fabricated with one wide bandgap polymer D18-Cl as donor, and well compatible Y6 and Y6-1O as acceptor. The open-circuit-voltage (*V_OC_*) of ternary OPVs is monotonously increased along with the incorporation of Y6-1O, indicating that the alloy state should be formed between Y6 and Y6-1O due to their excellent compatibility. The energy loss can be minimized by incorporating Y6-1O, leading to the *V_OC_* improvement of ternary OPVs. By finely adjusting the Y6-1O content, a power conversion efficiency of 17.91% is achieved in the optimal ternary OPVs with 30 wt% Y6-1O in acceptors, resulting from synchronously improved short-circuit-current density (*J_SC_*) of 25.87 mA cm^−2^, fill factor (FF) of 76.92% and *V_OC_* of 0.900 V in comparison with those of D18-Cl : Y6 binary OPVs. The *J_SC_* and FF improvement of ternary OPVs should be ascribed to comprehensively optimal photon harvesting, exciton dissociation and charge transport in ternary active layers. The more efficient charge separation and transport process in ternary active layers can be confirmed by the magneto-photocurrent and impedance spectroscopy experimental results, respectively. This work provides new insight into constructing highly efficient ternary OPVs with well compatible Y6 and its derivative as acceptor.

## Teaser

By employing the well compatible Y6 : Y6-1O as acceptor and wide bandgap polymer D18-Cl as donor, the PCE of 17.91% is achieved in optimized ternary OPVs, resulting from the simultaneously improved *J_SC_*, FF and *V_OC_*.

## INTRODUCTION

In recent years, remarkable progress has been made on single junction organic photovoltaics (OPVs) due to the flourishing development of materials and device engineering, leading to over 17% power conversion efficiency (PCE) [1–4]. The organic semiconductor materials usually possess relatively narrow absorption spectra with the full-width at half-maximum less than 150 nm, resulting in limited photon harvesting in traditional binary active layers. Ternary OPVs have been established to improve photon harvesting of active layers as much as possible by integrating two donors/one acceptor or one donor/two acceptors with complementary absorption spectra into one active layer. In fact, it is dubious to warrant performance improvement of ternary OPVs by taking the selection rule simply based on the absorption spectral complementarity among the used materials. Charge traps are ineluctably introduced in ternary active layers due to the different lowest-unoccupied-molecular-orbital (LUMO) energy levels between two acceptors or the distinct highest-occupied-molecular-orbital (HOMO) energy levels between two donors. The energy levels of the used materials should be considered to prevent forming deep charge traps in ternary active layers. Meanwhile, the incorporation of a third component with appropriate energy levels can help achieve relatively high open-circuit-voltage (*V_OC_*) in comparison with the host binary system, resulting in minimized energy loss (*E_loss_*) of OPVs. It should be kept in mind that morphology optimization of active layers is a major challenge for the preparation of efficient ternary OPVs because of their distinct chemical and physical properties. The good material compatibility should provide larger potential to subtly adjust the phase separation degree of active layers, where the third component will take a critical role as morphology regulator. The optical bandgap, energy level and compatibility among the used materials should be comprehensively deliberated to develop efficient ternary OPVs. The appropriate third component can usually play multiple roles in the performance improvement of OPVs, such as improving photon harvesting [5,6], modifying molecular crystallinity [7], minimizing energy loss [8–10], adjusting photogenerated exciton distribution [11], and ameliorating phase separation degree for efficient exciton dissociation and charge transport [12–14]. Nowadays, most of the representative efficient OPVs were fabricated with Y6 or its derivatives as acceptor, which possess a novel A-DA^′^D-A structure with relatively low bandgap. The outstanding efficiencies of Y6 or its derivative-based OPVs should be mainly attributed to their wide and high photoresponsivity with relatively low *E_loss_* (0.5–0.6 eV) [15–18]. The Y6 derivatives are usually designed and synthesized by slightly tailoring the function groups of Y6, resulting in slightly varied optical bandgap and energy level, as well as good compatibility with Y6. The well compatible Y6 and its derivatives may prefer to form an alloyed state for efficient electron transport in ternary active layers. The superiorities of Y6 and its derivatives may be simultaneously combined into one cell by employing their blends as acceptor. Highly ternary OPVs based on Y6 and its derivatives as acceptors are still rarely reported. In this work, a PCE of 17.91% is achieved in ternary OPVs with Y6 : Y6-1O as acceptor and wide bandgap polymer D18-Cl as donor. The PCE of 17.91% should be among the highest values of all ternary OPVs.

The chemical structures of D18-Cl, Y6 and Y6-1O are exhibited in Fig. S1a. The similar chemical structures of Y6 and Y6-1O suggest their good compatibility, which is beneficial in forming an alloy state for efficient electron transport in active layers. The absorption spectra of neat films and blend films were measured and are shown in Fig. S1b and c, respectively. The characteristic absorption peaks of D18-Cl, Y6 and Y6-1O are located at ∼575, ∼830 and ∼800 nm, respectively. The photon harvesting of ternary blend films is gradually increased in the short wavelength range and decreased in the long wavelength range along with Y6-1O incorporation, which can be well explained by the absorption spectra of corresponding neat films. The well optimal photon harvesting of ternary blend films should be achieved by adjusting the Y6-1O content. All binary and ternary OPVs were fabricated with a conventional configuration of ITO/PEDOT:PSS/Active layer/PDIN/Al. The upside-down solvent vapor annealing treatment was employed with carbon disulfide for 20 s on active layers to optimize the phase separation degree. The detailed experimental procedure is described in the Supporting Information. The 17.07% PCE is achieved in D18-Cl : Y6 binary OPVs, which is larger than that of 15.44% for D18-Cl : Y6-1O binary OPVs. The Y6-1O binary OPVs exhibit a relatively large *V_OC_* of 0.929 V compared with that of 0.881 V for D18-Cl : Y6 binary OPVs, which helps to improve the *V_OC_*s of ternary OPVs. The PCE of ternary OPVs arrives at 17.91% by incorporating 30 wt% Y6-1O, resulting from a simultaneously improved short-circuit current-density (*J_SC_*) of 25.87 mA cm^−2^, fill factor (FF) of 76.92% and *V_OC_* of 0.900 V compared with those of D18-Cl : Y6 binary OPVs. The performance improvement of ternary OPVs should be attributed to enhanced photon harvesting, minimized energy loss and improved active layer morphology for efficient exciton dissociation and charge transport in ternary active layers. This work may provide a new viewpoint for fabricating highly efficient ternary OPVs based on well compatible non-fullerene materials as acceptor.

## RESULTS AND DISCUSSION

**Figure 1.**
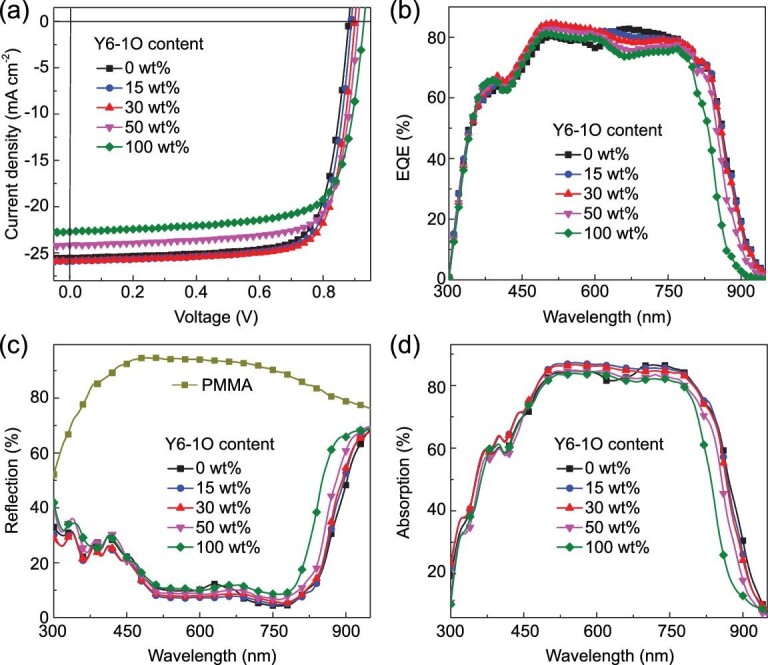
(a) The *J − V* curves of typical OPVs with different Y6-1O content. (b) EQE spectra of the corresponding OPVs. (c) The reflection spectra of corresponding OPVs and the special device. (d) Absorption spectra of active layers in corresponding OPVs.

The current-density versus voltage (*J* − *V*) curves of all OPVs were measured under AM 1.5G simulated solar light with 100 mW cm^−2^ intensity, as exhibited in Fig. 1a. Based on the *J* − *V* curves, key photovoltaic parameters of all OPVs are summarized in Table 1. The D18-Cl : Y6-based OPVs exhibit a PCE of 17.07% with a *J_SC_* of 25.53 mA cm^−2^, an FF of 75.88% and a *V_OC_* of 0.881 V. The D18-Cl : Y6-1O-based OPVs exhibit a PCE of 15.44% with a *J_SC_* of 22.72 mA cm^−2^, an FF of 73.14% and a *V_OC_* of 0.929 V. Both binary OPVs exhibit the FFs more than 73%, indicating that an efficient charge transport channel should be formed in two kinds of active layers. Meanwhile, there is apparent complementarity between *J_SC_*s and *V_OC_*s of two binary OPVs. The *J_SC_* of D18-Cl : Y6 binary OPVs is much higher than that of Y6-1O-based binary OPVs, and the *V_OC_* of D18-Cl : Y6-1O binary OPVs is much higher than that of Y6-based binary OPVs. The superiorities of two binary OPVs may be combined into one cell by employing Y6 : Y6-X as acceptor. Series of ternary OPVs were prepared by adjusting the content of Y6-1O in acceptors. A PCE of 17.91% is achieved in the optimal ternary OPVs with 30 wt% Y6-1O, resulting from a simultaneously improved *J_SC_* of 25.87 mA cm^−2^, FF of 76.92% and *V_OC_* of 0.900 V in comparison with those of D18-Cl : Y6 binary OPVs. The PCE of 17.91% is among the highest values for ternary OPVs. It should be highlighted that the 16.74% PCE and 76.07% FF can still be obtained in the ternary OPVs with 50 wt% Y6-1O in acceptors, indicating the excellent tolerance between Y6 and Y6-1O for fabricating efficient ternary OPVs. To better understand FF variation of ternary OPVs dependence on Y6-1O content in acceptors, the series resistance (*R_S_*) and shunt resistance (*R_SH_*) were calculated according to *J − V* curves of OPVs, as summarized in Table 1. The *R_S_*s of OPVs show a decreased and then increased trend along with the increase of Y6-1O content, and the *R_SH_*s of OPVs exhibit an opposite trend compared with *R_S_*s. The minimum *R_S_* of 2.54 Ω cm^2^ and maximum *R_SH_* of 925.9 Ω cm^2^ were simultaneously obtained for the OPVs with 30 wt% Y6-1O in acceptors, which are responsible for the highest FF of the optimal ternary OPVs.

**Table 1.** Key photovoltaic parameters of all OPVs with different Y6-1O content.

Y6-1O content	*J_SC_*	*V_OC_*	FF	PCE (Ave. ± Dev.^a^)	*R_S_*	*R_SH_*
(wt%)	(mA cm^−2^)	(V)	(%)	(%)	(Ω cm^2^)	(Ω cm^2^)
0	25.53	0.881	75.88	17.07 (16.86 ± 0.21)	2.91	841.8
15	25.89	0.889	76.27	17.55 (17.37 ± 0.23)	2.69	863.6
30	25.87	0.900	76.92	17.91 (17.83 ± 0.21)	2.54	925.9
50	24.16	0.911	76.07	16.74 (16.49 ± 0.27)	2.87	881.8
100	22.72	0.929	73.14	15.44 (15.26 ± 0.19)	3.16	626.0

^a^Average and deviation (Ave. ± Dev.) of PCEs were calculated with 10 individual cells from different batches.

To clarify the contribution of Y6-1O on performance improvement, the external quantum efficiency (EQE) spectra of OPVs were measured and are shown in Fig. 1b. It is apparent that EQE values of the optimal ternary OPVs can be improved in the short wavelength range from 370 to 630 nm, which may be mainly caused by well-balanced photon harvesting, exciton dissociation, charge transport and collection. The EQE values in the long wavelength range are gradually decreased along with the incorporation of Y6-1O, which can be reasonably explained by the relatively low EQE spectra of D18-Cl : Y6-1O binary OPVs. For a better understanding of the variation of EQE spectra dependence on Y6-1O content in acceptors, the absorption spectra of the active layers in real cells were investigated according to the reflection spectra of corresponding cells. The reflection spectra and absorption spectra of corresponding cells are shown in Fig. 1c and d, respectively. A special device with a structure of ITO/PEDOT:PSS/PMMA/PDIN/Al was fabricated to eliminate the effect of parasitic absorption in cells. The light absorption of glass/ITO substrate and interfacial layers can be obtained from this special device due to the negligible absorption of poly(methylmethacrylate) (PMMA) in the whole wavelength range. The absorption spectra of active layers in cells can be calculated by subtracting the parasitic absorption from the total absorption in OPVs. The detailed calculation process is described in the Supporting Information. The absorption spectra of the active layers in cells can really reflect the contribution of the third component to the photon harvesting of ternary active layers by considering the interference effect between the incident light and the reflected light from the Al electrode. The slightly decreased EQE values of the optimal ternary OPVs in the long wavelength range should be ascribed to decreased photon harvesting of ternary active layers. The photon harvesting of optimal ternary active layers is enhanced in the short wavelength range from 370 to 630 nm compared with that of binary active layers, which may be attributed to the more ordered molecular arrangement of D18-Cl and Y6 in ternary active layers with appropriate Y6-1O incorporation. The Y6-1O may act as morphology regulator to adjust molecular arrangement of D18-Cl and Y6 in ternary active layers.

To further investigate the reasons behind the performance improvement by incorporating Y6-1O, the detailed *E_loss_* of OPVs was investigated. The *E_loss_* of OPVs can be calculated according to the equation: *E_loss__ _*= *E_g_* − e*V_OC_*, in which the bandgap (*E_g_*) can be estimated according to the intersection between the normalized emission and absorption spectra of the acceptor films [19], as shown in Fig. S2. The total *E_loss_* of OPVs can be divided into three parts, as described in the following equation:

$$\begin{eqnarray*} {E_{l\!o\!s\!s}} &=& {E_g}{\rm{\ }} - e {V_{OC}} = {\rm{\ }}\Delta {E_1} + \Delta {E_2}+ \Delta {E_3}\nonumber\\ &=& \left( {{E_g} - eV_{OC}^{SQ}} \right){\rm{\ }}+ \left( {eV_{OC}^{SQ} {\ - \ } eV_{OC}^{Rad}} \right)\nonumber\\ && + \left( {eV_{OC}^{Rad} {\ - \ } e{V_{OC}}} \right)\!.\end{eqnarray*}$$

**Figure 2.**
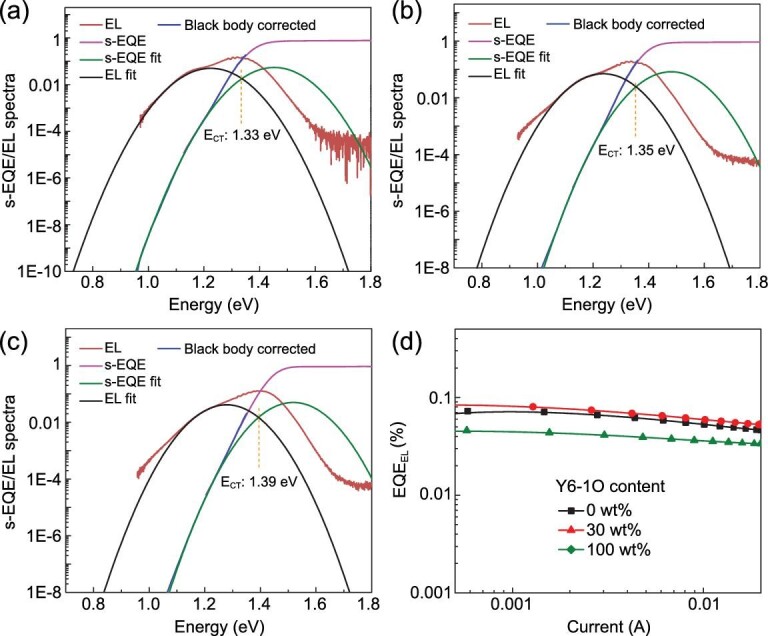
The s-EQE and EL spectra of (a) D18-Cl : Y6, (b) the optimal ternary and (c) D18-Cl : Y6-1O-based OPVs. (d) The EQE_EL_ spectra of typical binary and ternary OPVs.

Here, $\Delta {E_1}$, $\Delta {E_2}$ and $\Delta {E_3}$ represent unavoidable radiative recombination loss above the bandgap, radiative recombination loss below the bandgap and non-radiative recombination loss, respectively. $V_{OC}^{SQ}$ and $V_{OC}^{Rad}$ are voltages in Shockley-Queisser limit and radiative limit, respectively [20]. The highly sensitive EQE (s-EQE) spectra and electroluminescence (EL) spectra were measured to evaluate the specific *E_loss_* of OPVs, as shown in Fig. 2a–c. $V_{OC}^{SQ}$ is determined by ${E_{CT}}$/*q*, where ${E_{CT}}$ represents the energy of charge transfer state. ${E_{CT}}$ can be estimated by fitting the lower energy part of s-EQE spectra and higher energy part of EL spectra based on Marcus theory. According to the ${E_{CT}}$ values, the $V_{OC}^{SQ}$ is calculated to be 1.072, 1.090 and 1.128 V, corresponding to $\Delta {E_1}$ of 0.383, 0.367 and 0.359 eV for D18-Cl : Y6, and the optimal ternary- and D18-Cl : Y6-1O-based OPVs, respectively. The $\Delta {E_1}$ of the optimal ternary OPVs is lower than that of D18-Cl : Y6-based binary OPVs, which contributes the performance improvement of ternary OPVs. The $V_{OC}^{Rad}$ can be obtained according to the following equation:

$$\begin{eqnarray*}q V_{OC}^{Rad} = \ KTln\! \left( {\frac{{{J_{ph}}\left( {{V_{oc}}} \right)}}{{{J_{0,\!\ rad}}}} + 1} \right)\!.\end{eqnarray*}$$

Here, ${J_{0, rad}}$ is the saturation current density calculated by considering only the blackbody radiation of the cell with the real absorption profile. *K*, *T* and *q* represent Boltzmann's constant, temperature of the sample and elementary charge, respectively. ${J_{ph}}( {{V_{oc}}} )$ is the photocurrent at open-circuit condition [21]. The calculated $\Delta {E_2}\ $of optimal ternary OPVs is slightly increased compared with binary OPVs, as summarized in Table S1. $\Delta {E_3}$ is linearly related to the natural logarithm of EQE_EL_, as described by $\Delta {\rm{ }}{E_3} = {\rm{ }} -\!\! KTln( {\rm EQE_{EL}} )$. As shown in Fig. 2d, the optimized ternary OPVs show higher EQE_EL_ of 9.96 × 10^−4^ than that of two binary OPVs, leading to the reduced non-radiative loss of 0.175 eV. The total ${E_{l\!o\!s\!s}}$ can be obtained according to the sum of $\Delta {E_1}$, $\Delta {E_2}$ and $\Delta {E_3}$. The *E_loss_* of optimal ternary OPVs is 0.582 eV, which is lower than that of 0.597 eV in D18-Cl : Y6-based binary OPVs. The reduced *E_loss_* by incorporating Y6-1O should play a vital role in the performance improvement of ternary OPVs.

**Figure 3.**
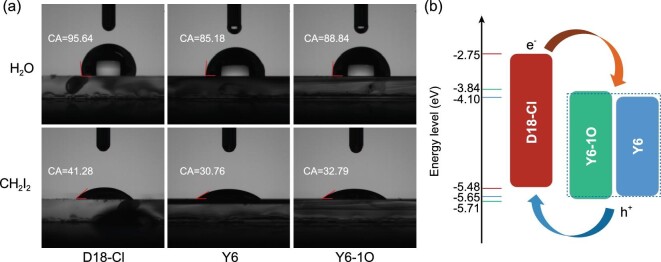
(a) The contact angle images of neat D18-Cl, Y6 and Y6-1O films. (b) Energy levels of used materials and diagram of alloy-like state.

For the OPVs with the same interfacial layers and electrodes, the *V_OC_* strongly depends on the energy level difference between the donor's HOMO and acceptor's LUMO. The photogenerated holes will be transported along the channels formed by D18-Cl with the constant HOMO energy level. The *V_OC_*s of ternary OPVs are monotonously increased along with Y6-1O incorporation, which should be ascribed to the gradually evaluated LUMO energy levels of an alloyed state of Y6 : Y6-1O [22,23]. To evaluate the compatibility of the used materials, the contact angles measurement of neat D18-Cl, Y6 and Y6-1O films were carried out based on liquid drops of water and methylene iodide, as shown in Fig. 3a. According to Wu's model [24–26], the surface energy (*γ*) values of D18-Cl, Y6 and Y6-1O films are calculated to be 39.8, 43.9 and 43.2 mN m^−1^, respectively. The similar *γ* values of 43.9 and 43.2 mN m^−1^ indicate good compatibility between Y6 and Y6-1O. According to the *γ* values of neat films, the interfacial energy between two different materials can be calculated by employing the following equation:

$$\begin{eqnarray*}{\gamma _{X:Y}} &=& {\gamma _X}\ + {\gamma _Y} - \frac{{4\gamma _X^d\gamma _Y^d}}{{\gamma _X^d + \gamma _Y^d}}- \frac{{4\gamma _X^p\gamma _Y^p}}{{\gamma _X^p + \gamma _Y^p}}.\end{eqnarray*}$$

Here, ${\gamma _{X: Y}}$ represents the interfacial energy between material *X* and *Y*;$\ {\gamma _X}$ and ${\gamma _Y}$ represent the surface energy of the neat materials; and superscript *d* and *p* are the dispersion and polar components calculated according to the contact angles, respectively [27]. The interfacial energy between Y6 and Y6-1O is calculated to be 0.16 mN m^−1^, which is much lower than that (${\gamma _{D18 - X: Y6}}\ $= 0.68 mN m^−1^, ${\gamma _{D18 - X: Y6 - X}}\ $= 1.37 mN m^−1^) between donor and acceptor. The excellent compatibility between Y6 and Y6-1O can be firmly confirmed by the rather low interfacial energy, which is conducive to forming an alloy state of Y6 : Y6-1O. To further investigate the intermolecular interaction between Y6 and Y6-1O, photoluminescence (PL) spectra of Y6, Y6-1O and their blend films were measured and are shown in Fig. S3a. It is apparent that the PL emission peaks of blend films are gradually shifted from 860 to 850 nm along with the increase of Y6-1O content. Meanwhile, the PL emission intensity of the blend films is monotonously increased along with Y6-1O incorporation, suggesting the negligible charge and energy transfer between Y6 and Y6-1O. To further demonstrate the negligible charge transfer between two acceptors, a series of cells were fabricated with Y6, Y6-1O and Y6 : Y6-1O as active layers. The *J − V* curves of the cells were measured under AM 1.5G illumination with light intensity of 100 mW cm^−2^ and are shown in Fig. S3b. The *J_SC_* of Y6 : Y6-1O-based cells is in between that of Y6-based and Y6-1O-based cells, suggesting the negligible charge transfer or exciton dissociation at Y6 and Y6-1O interfaces. According to the above analysis, the dynamic processes in ternary OPVs can be schematically depicted in Fig. 3b. The photogenerated holes will be transported along the channels formed by D18-Cl, and the photogenerated electrons will be transported along the channels formed by Y6 : Y6-1O. The bi-continuous charge transport channels should be well formed in ternary active layers, resulting in over 76% FF for all ternary OPVs.

**Figure 4.**
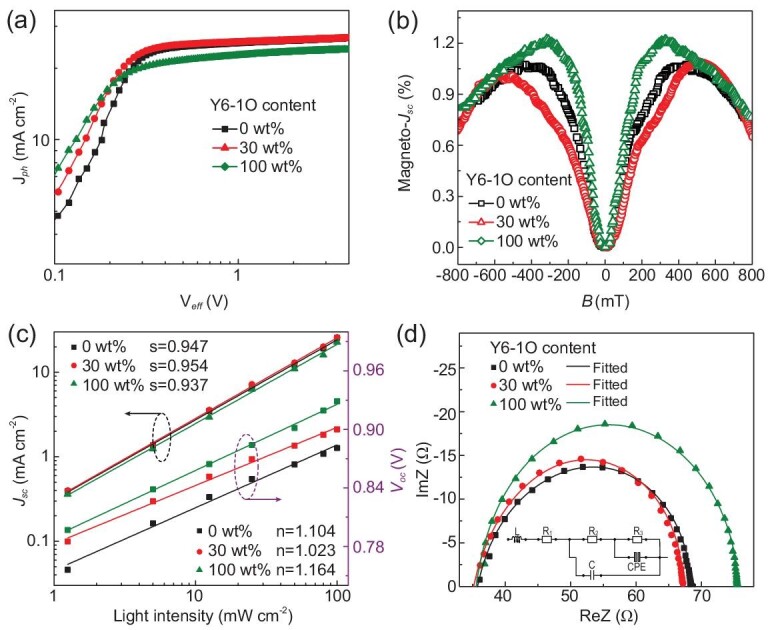
(a) *J_ph_ − V_eff_* curves of typical OPVs. (b) Magneto-photocurrent curves of typical OPVs under 635 nm photo-excitation. (c) *J_SC_* − *P_light_* and *V_OC_* − *P_light_* curves of typical OPVs. (d) Impedance spectra responses of corresponding OPVs; the inset represents the equivalent circuit model.

To investigate charge generation and exciton dissociation properties in active layers, photocurrent density (*J_ph_*) versus effective voltage (*V_eff_*) characteristics of typical OPVs were measured and are shown in Fig. 4a. Assuming that all photogenerated excitons are dissociated into free charges and then swept out under relatively large *V_eff_* conditions, the *J_ph_* can be defined as the saturated photocurrent density (*J_sat_*) [28–31]. The *J_sat_* values are 26.78, 26.92 and 23.96 mA cm^−2^ for D18-Cl : Y6 binary, optimal ternary and D18-Cl : Y6-1O binary OPVs. The relatively large *J_sat_* value of the optimal ternary OPVs indicates improved photogenerated exciton density in ternary active layers. The exciton dissociation efficiency and charge collection efficiency can be evaluated according to J_ph_^*^ /*J_sat_* and *J_ph_^#^* /*J_sat_*, respectively. Here, J_ph_^*^ and *J_ph_^#^* represent the *J_ph_* values under short-circuit and maximal power output conditions, respectively. The J_ph_^*^ /*J_sat_* and *J_ph_^#^* /*J_sat_* values of OPVs are increased to 96.1% and 86.5%, respectively, by incorporating 30 wt% Y6-1O in acceptors. The increased J_ph_^*^ /*J_sat_* and *J_ph_^#^* /*J_sat_* values of ternary OPVs suggest that the phase separation of active layers can be well optimized by incorporating appropriate Y6-1O as morphology regulator, leading to improved FF of 76.92% for the optimal ternary OPVs. The detailed *J_ph_* values of typical OPVs at different conditions are summarized in Table S2. To gain more insight into the charge separation process in distinct active layers, the magneto-photocurrent (magneto-*J_SC_*) characteristics of typical OPVs were investigated. The magneto-*Jsc* was employed to monitor the charge separation at donor–acceptor interfaces based on the following processes. The singlet excitons are generated due to the spin selection rule under photoexcitation. Singlet excitons can migrate to the donor–acceptor interface to produce electron-hole pairs. The electron-hole pairs can be partially converted into triplet polaron pairs through intersystem crossing caused by internal magnetic interaction such as hyperfine interaction and spin-orbital coupling, leading to coexistence of singlet and triplet electron-hole pairs. The populations of singlet and triplet electron-hole pairs can be changed with the application of magnetic field through intersystem crossing, leading to a varied photocurrent due to different dissociation rates of singlets and triplets. The magneto-*J_SC_* of OPVs is firmly related to the ratio of spin-related singlet state and triplet state forming at donor–acceptor interfaces. The magneto-*J_SC_* was recorded by measuring the photocurrent of OPVs as a function of magnetic field under the 635 nm photoexcitation condition, as shown in Fig. 4b. The magneto-*J_SC_* can be calculated according to the following equation:

$$\begin{equation*}
{\rm{magneto}} - {J_{\!SC}}{\rm{\ }} = \frac{{I\! \!\left( {\rm{B}} \right) - I\! \!\left( 0 \right)}}{{I\!\! \left( 0 \right)}}\ \times \ 100\%.
\end{equation*}$$

Here, *I*(B) and *I*(0) represent the photocurrent measured with and without a magnetic field, respectively [32,33]. As shown in Fig. 4b, the optimal binary and ternary OPVs show a positive magneto-*J_SC_* signal within the sweeping fields ± 800 mT, which should be ascribed to the increased singlet/triplet ratio under a magnetic field. In comparison with binary OPVs, the optimal ternary OPVs exhibit the relatively small magnitude of magneto-*J_SC_* at low magnetic field strength. The relatively small magneto-*J_SC_* of the optimal ternary OPVs suggests more efficient charge separation in ternary active layers, which can well support the increased *J_SC_* of the optimal ternary OPVs.

To understand the charge recombination process in active layers, *J − V* curves of typical OPVs were measured under different light intensity, as shown in Fig. S4. Based on the *J − V* curves, *J_SC_* and *V_OC_* of typical OPVs versus the incident intensity (*P_light_*) were plotted as shown in Fig. 4c. The relationship between *J_SC_* and *P_light_* can be described as *J_SC_* ∝ *P_light_^s^* [34–37]. The power-law exponential factor *s* represents the degree of bimolecular recombination, which should be equal to 1 if the bimolecular recombination is completely suppressed. The fitted *s* values are 0.947, 0.954 and 0.937 for D18-Cl : Y6 binary, optimal ternary and D18-Cl : Y6-1O binary OPVs. The fitted *s* value of the optimal ternary OPVs is much closer to 1, indicating that the bimolecular recombination in active layers can be effectively restrained with appropriate Y6-1O incorporation. The degree of trap-assisted recombination in OPVs can be evaluated by applying the equation *V_OC_* ∝ *n*(*KT/q*)ln*P_light_*, in which *K*, *T* and *q* represent the Boltzmann constant, absolute temperature and elementary charge, respectively [38–41]. The trap-assisted recombination in active layers should be negligible with the slope close to *KT*/*q*. The slope is decreased from 1.104 *KT*/*q* (D18-Cl : Y6 binary OPVs) or 1.164 *KT*/*q* (D18-Cl : Y6-1O binary OPVs) to 1.023 *KT*/*q* for the optimal ternary OPVs, indicating that trap-assisted recombination can be suppressed by incorporating appropriate Y6-1O. The synchronously mitigated bimolecular and trap-assisted recombination of the optimal ternary active layers is conducive to efficient charge transport and collection, leading to improved FF of 76.92% for the optimal ternary OPVs.

To gain more insight into the effect of incorporating Y6-1O into the charge transport and recombination process, the impedance spectroscopy (IS) measurements were carried out in frequency range from 20 Hz to 3.5 MHz. Figure 4d exhibits the Nyquist plots of the typical binary and ternary OPVs measured at V = *V_OC_*. The equivalent circuit model used to fit Nyquist plots data are shown in the inset of Fig. 4d. In this model, the inductance (*L*) was employed to eliminate the impact of the connecting wires during high-frequency scans; *R_1_* represents the series resistance deriving from electrodes and bulk resistance in active layers; and *R_2_* and *R_3_* represent transport and recombination resistance in active layers, respectively [42]. The capacitor (*C*) accounts for the dielectric behavior of OPVs. The constant phase element (CPE) is introduced to the circuit model, which indicates non-ideal behavior of the capacitor. The CPE is defined by the equation *Z* = (*CPE_T_*)^−1^(*iw*)$^{{C\!P\!E}_P}$, in which *w* is the angular frequency, and *CPE_T_* and *CPE_P_* represent capacitance and inhomogeneous constant, respectively [43,44]. If *CPE_P_* is equal to 1, the *CPE* is identical to an ideal capacitor without the presence of any defects at the interface between donor and acceptor [45]. The fitted parameters of typical OPVs are summarized in Table S3. The *R_2_* values are decreased from 23.2 (D18-Cl : Y6 binary OPVs) or 32.3 (D18-Cl : Y6-1O binary OPVs) to 18.1 Ω for the optimal ternary OPVs, while the *R_3_* values are increased from 11.3 (D18-Cl : Y6 binary OPVs) or 9.29 (D18-Cl : Y6-1O binary OPVs) to 16.2 Ω for the optimal ternary OPVs. The smaller *R_2_* and larger *R_3_* values of the optimal ternary OPVs should facilitate charge transport and restrain charge recombination in ternary active layers, leading to improved FF for the optimal ternary OPVs. The *CPE_P_* values are 0.941, 0.977 and 0.956 for D18-Cl : Y6 binary, optimal ternary and D18-Cl : Y6-1O binary OPVs, respectively. In comparison with two binary OPVs, the *CPE_P_* of the optimal ternary OPVs is much closer to 1, indicating that the interfacial capacitance is more electrically ideal by incorporating appropriate Y6-1O.

The charge mobility in blend films were characterized based on the space charge limited current (SCLC) method. The configurations of hole-only and electron-only devices are described in the Supporting Information. The ln(*Jd^3^/V^2^*)-(*V/d*)^0.5^ curves of hole-only and electron-only devices are depicted in Fig. S5. The hole mobility (*μ_h_*) and electron mobility (*μ_e_*) of blend films are increased to 8.67 × 10^−4^ cm^2^ V^−1^ s^−1^ and 6.06 × 10^−4^ cm^2^ V^−1^ s^−1^ by incorporating 30 wt% Y6-1O in acceptors, indicating that a more efficient charge transport channel should be formed in the optimal ternary active layers. The *μ_h_*/*μ_e_* values can be employed to estimate charge transport balance in active layers [46–49]. The *μ_h_*/*μ_e_* values are 1.63, 1.43 and 1.70 for D18-Cl : Y6 binary, optimal ternary and D18-Cl : Y6-1O binary OPVs. The *μ_h_*/*μ_e_* value of optimal ternary OPVs is closest to 1, indicating a more balanced charge transport in the optimal ternary active layers. The increased charge mobility and more balanced charge transport can rationalize the relatively high FF of 76.92% in the optimal ternary OPVs. The detailed *μ_h_*, *μ_e_*, and *μ_h_*/*μ_e_* of the corresponding devices are summarized in Table S2. It is well known that charge transport is closely related to the molecular packing property of blend films. The grazing-incidence wide-angle X-ray scattering (GIWAXS) characterization was performed to investigate the effect of Y6-1O content on molecular arrangement.

**Figure 5.**
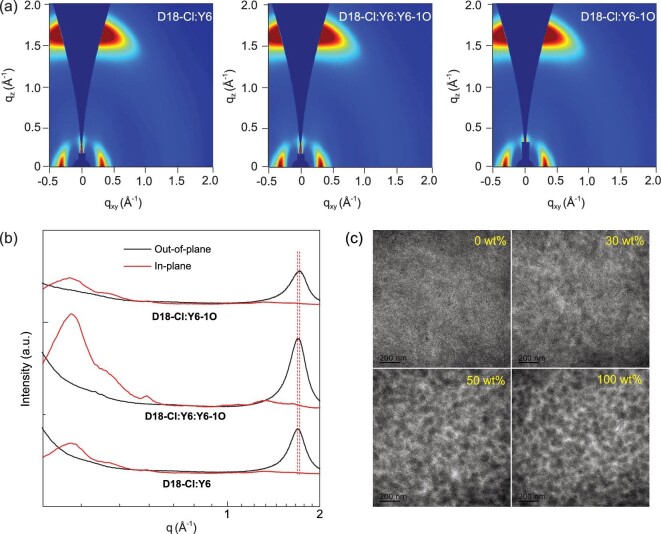
(a) The 2D-GIWAXS patterns of corresponding blend films. (b) The out-of-plane (black line) and in-plane (red line) line-cut profiles of the GIWAXS patterns in blend films. (c) TEM images of blend films with distinct Y6-1O content.

The 2D GIWAXS patterns and the corresponding out-of-plane (OOP) and in-plane (IP) profiles of neat D18-Cl, Y6 and Y6-1O films are shown in Fig. S6. The neat Y6 and Y6-1O films exhibit relatively apparent OOP (010) and IP (100) diffraction peaks, suggesting preferential face-on orientation with respect to the substrates. Except for the OOP (010) and IP (100) diffraction peaks, neat D18-Cl films also show relatively apparent OOP (100) diffraction peak, signifying the coexistence of face-on and edge-on orientation with respect to the substrates. The 2D GIWAXS patterns and the corresponding OOP and IP profiles of blend films are shown in Fig. 5a and b, respectively. Upon blending D18-Cl with Y6 or Y6-1O, the resultant blend films exhibit a preferential face-on orientation, which is evidenced by the pronounced OOP (010) and IP (100) diffraction peaks. The OOP (010) diffraction peaks of binary blend films are located at the same positions as in their corresponding neat acceptor films, suggesting that the π–π stacking in blend films is dominated by acceptors. The IP (100) diffraction peaks of blend films are located at 0.31 Å^−1^, which should be mainly attributed to the ordered lamellar stacking of D18-Cl, as confirmed by the relatively strong IP (100) diffraction peak of neat D18-Cl film. For the optimal ternary blend films, the OOP (010) and IP (100) diffraction peaks are simultaneously improved in comparison with those of two binary blend films, suggesting more ordered face-on orientation in the optimal ternary blend films. The π–π stacking distance in the optimal ternary blend films is calculated to be 3.67 Å, which is slightly shorter than that of D18-Cl : Y6-based blend films. The reduction of the intermolecular π–π stacking distance in OOP direction should promote the electron transport in ternary active layers. The molecular correlation length (*L_C_*) can be evaluated according to the equation *L_C_* = 2π*k*/*f_whm_*, where *k* is the shape factor typically with the value of 0.9, and *f_whm_* is the full-width at half-maximum of a diffraction peak [50]. In the optimal ternary blend films, the *L_C_* related to lamellar stacking is calculated to be 5.8 nm, which is larger than that of binary blend films. The increased *L_C_* in ternary blend films corresponds to the higher D18-Cl domain crystallinity. In comparison with binary blend films, the quite different IP (100) diffraction peak in ternary blend films should originate from more ordered lamellar stacking and higher crystallinity of D18-Cl. The more ordered molecular arrangement of D18-Cl should facilitate hole transport, leading to increased hole mobility of ternary active layers. The detailed vector values of diffraction peaks and *L_C_* values in neat and blend films are summarized in Table S4.

To unveil the effect of the Y6-1O content on the bulk morphology of blend films, transmission electron microscopy (TEM) was carried out on the blend films, as shown in Fig. 5c. The homogeneous and nanofibrous structures can be observed from the TEM images of Y6-based binary blend films. The island-like structure with a relatively large phase separation degree can be observed from the TEM images of Y6-1O-based binary blend films, which can be well explained by the relatively large interfacial energy between D18-Cl and Y6-1O. As observed from the TEM images of ternary blend films, the aggregated structure between donor and acceptor, as well as the phase separation degree are gradually varied along with the incorporation of Y6-1O. The gradually varied morphology of ternary blend films depending on Y6-1O content suggests that Y6-1O can act as morphology regulator. The appropriate phase separation degree should be formed in the optimal ternary active layers, which provide enough donor–acceptor interfacial area for exciton dissociation, as well as a bi-continuous percolating charge transport path to electrodes.

## CONCLUSION

The PCE of 17.07% is achieved in D18-Cl : Y6-based OPVs. On this basis, a series of ternary OPVs are fabricated by employing Y6-1O as the third component. The 17.91% PCE is achieved in the optimal ternary OPVs with 30 wt% Y6-1O in acceptors, which should be among the highest levels in ternary OPVs. The photon harvesting, molecular arrangement and phase separation degree in active layers can be finely adjusted by incorporating appropriate Y6-1O, leading to the synchronously improved *J_SC_* and FF of ternary OPVs. The improved phase separation and molecular arrangement are beneficial to efficient charge separation and transport in ternary active layers, which can be confirmed by the experimental results of GIWAXS and TEM. The more efficient charge separation and transport processes in ternary active layers can be well explained by the magneto-*J_SC_* and IS measurement results. Meanwhile, the *E_loss_* of ternary OPVs is gradually decreased along with the incorporation of Y6-1O, leading to improved *V_OC_* in ternary OPVs. This work delivers more insight into the potential reasons for the third component’s effect on performance improvement of ternary OPVs.

## Supplementary Material

nwaa305_Supplemental_File
