# Supplementary material for: Approaching 18% efficiency of ternary organic photovoltaics with wide bandgap polymer donor and well compatible Y6 : Y6-1O as acceptor
Source: Natl Sci Rev. 2020 Dec 31;8(8):nwaa305. doi: 10.1093/nsr/nwaa305 (PMC8363335; doi:10.1093/nsr/nwaa305)
Supplement: nwaa305_Supplemental_File [file nwaa305_supplemental_file.docx]

**Approaching 18% efficiency of ternary organic photovoltaics with wide bandgap polymer donor and well compatible Y6:Y6-1O as acceptor**

Xiaoling Ma ^a,†^, Anping Zeng ^b,†^, Jinhua Gao ^a^, Zhenghao Hu ^a^, Chunyu Xu ^a^, Jae Hoon Son ^d^, Sang Young Jeong ^d^, Caixia Zhang ^a^, Mengyang Li ^e^, Kai Wang ^a^, He Yan ^b,c^, Zaifei Ma ^e^, Yongsheng Wang ^a^, Han Young Woo ^d,^*and Fujun Zhang ^a,^*

^a^Key Laboratory of Luminescence and Optical Information, Ministry of Education, Beijing Jiaotong University, Beijing 100044, China

^b^Department of Chemistry, The Hong Kong University of Science and Technology, Hong Kong, China

^c^Institute of Polymer Optoelectronic Materials and Devices, State Key Laboratory of Luminescent Materials and Devices, South China University of Technology, Guangzhou 510640, China
^d^Organic Optoelectronic Materials Laboratory, Department of Chemistry, College of Science, Korea University, Seoul 02841, Republic of Korea

^e^Center for Advanced Low-dimension Materials, State Key Laboratory for Modification of Chemical Fibers and Polymer Materials, College of Materials Science and Engineering, Donghua University, Shanghai 201620, China

∗**Corresponding authors.** E-mails: fjzhang@bjtu.edu.cn; hywoo@korea.ac.kr

^†^Equally contributed to this work.

**Experimental section**

Device Fabrication: The patterned indium tin oxide (ITO) coated glass substrates (10 Ω per square) were cleaned via sequential sonication in detergent, de-ionized and ethanol and then blow-dried by high-purity nitrogen. All pre-cleaned ITO substrates were treated by oxygen plasma for 1 minute (min) to improve their work function and clearance. Subsequently, poly(3,4-ethylenedioxythiophene):poly(styrene sulfonate) (PEDOT:PSS, purchased from H.C. Starck co. Ltd.) solution was spin-coated on ITO substrates at 5000 round per minute (RPM) for 40 s and dried at 150 °C for 10 min in atmospheric air. Then ITO substrates coated with PEDOT:PSS films were transferred into a high-purity nitrogen-filled glove box. The used D18-Cl and Y6-1O were designed and synthesized by He Yan’s group in Hong Kong University of Science and Technology, the two materials were purchased from the eFlexPV limited company owned by Yan. The Y6 was purchased from Solarmer Materials Inc. The D18-Cl:Y6 and D18-Cl:Y6-1O with 1:1.6 weight ratio were dissolved in chloroform to prepare 13 mg/ml binary blend solution. The proportions of Y6:Y6-1O are 1:0, 0.85:0.15, 0.7:0.3, 0.5:0.5, 0:1. The mixed solution was spin-coated onto the PEDOT:PSS/ITO substrates at 2500 RPM for 40 s to prepare the active layers. The active layers were up-side down solvent vapor annealed with carbon disulfide for 30 s. PDIN was dissolved in methanol with the addition of 0.25 vol% acetic acid to prepare a 2 mg mL^-1^ solution. The prepared PDIN solution was spin-coated onto the active layers at 5000 RPM for 40 s. Finally, aluminum (Al) electrode was deposited by thermal evaporation. For each of the organic photovoltaics (OPVs), the contacts were deposited at the vacuum condition of 10^−4^ Pa. The active area is approximately 3.8 mm^2^, which is defined by the overlapping area of ITO anode and Al cathode.

Device Measurement: The current density-voltage (*J-V*) curves of all the OPVs were measured by a Keithley 2400 unit in high-purity nitrogen-filled glove box. The AM 1.5G irradiation was provided by an XES-40S2 (SAN-EI ELECTRIC Co., Ltd) solar simulator (AAA grade, 70×70 mm^2^ photobeam size) with light intensity of 100 mW cm^-2^. Our simulator was corrected by using a standard monocrystalline silicon reference cell. The external quantum efficiency (EQE) spectra of OPVs were measured by a Zolix Solar Cell Scan 100. The absorption spectra of films were measured with a Shimadzu UV-3101 PC spectrometer. Transmission electron microscopy (TEM) images of blend films were obtained by a JEOL JEM-1400 transmission electron microscope operated at 80 kV. The configurations of hole-only and electron-only devices are ITO/PEDOT:PSS/Active layers/M_O_O_3_/Ag and ITO/ZnO/ Active layers/PDIN/Al, respectively. The reflection measurement of all devices was performed on a commercial QE measurement system (QE-RT3011, Enlitech) by using an integrating sphere. The absorption spectra of active layers were calculated by subtracting the parasitic absorptions (1-R_1_) from the total absorption in OPVs (1-R_2_), where R_1_ is the reflection spectrum of device ITO/PEDOT:PSS/PMMA/PDIN/Al, R_2_ is the reflection spectra of OPVs. The PMMA layer in the special cell is used to simulate the optical path in real OPVs because PMMA has negligible photon harvesting in the whole spectral range, and the light absorption of glass/ITO/PEDOT:PSS layers in real OPVs can be obtained from this special cell.


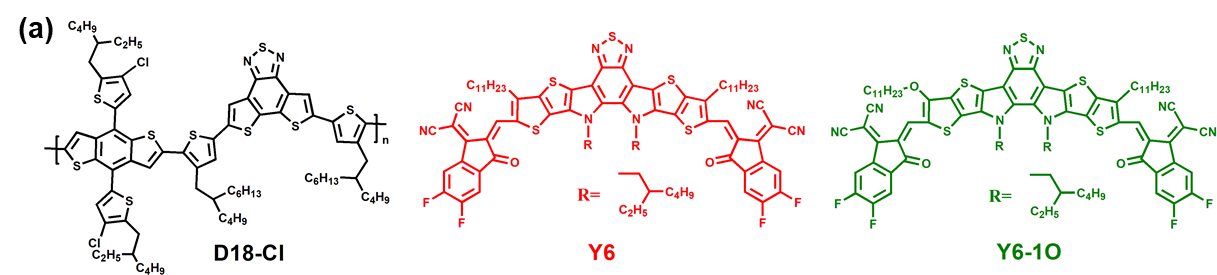

**Figure S1**. (a) Chemical structures of D18-Cl, Y6 and Y6-1O. (b) Normalized absorption spectra of neat films. (c) Absorption spectra of blend films with distinct Y6-1O content.

**Figure S2.** The normalized absorption and emission spectra of Y6, Y6-1O and Y6:Y6-1O blend films.

**Table S1**. Detailed *E_loss_* of the optimal binary and ternary OPVs.

| Y6-1O content  (wt%) | *E_g_*  (eV) | *E_CT_*  (eV) | e$V_{OC}^{SQ}$  (eV) | e$V_{OC}^{Rad}$  (eV) | $\Delta E_{1}$  (eV) | $\Delta E_{2}$  (eV) | $\Delta E_{3}$  (eV) | *E_loss_*  (eV) |
| --- | --- | --- | --- | --- | --- | --- | --- | --- |
| 0 | 1.455 | 1.330 | 1.072 | 1.038 | 0.383 | 0.034 | 0.180 | 0.597 |
| 30 | 1.457 | 1.350 | 1.090 | 1.050 | 0.367 | 0.040 | 0.175 | 0.582 |
| 100 | 1.487 | 1.390 | 1.128 | 1.095 | 0.359 | 0.033 | 0.195 | 0.587 |

**Figure S3.** (a) The PL spectra of Y6:Y6-1O blend films with different Y6-1O content under 700 nm light excitation. (b) *J-V* curves of cells with Y6, Y6-1O or Y6:Y6-1O as active layers.

**Table S2.** The key parameters of the optimized binary and ternary OPVs.

| Blend films | *J_ph_*^*^  (mA cm^-2^) | *J_ph_*^#^  (mA cm^-2^) | *J_sat_*  (mA cm^-2^) | *J_ph_*^*^/*J_sat_*  (%) | *J_ph_*^#^/*J_sat_*  (%) | *µ_h_*  (cm^2^ V^-1^ s^-1^) | *µ_e_*  (cm^2^ V^-1^ s^-1^) | *µ_h_/µ_e_* |
| --- | --- | --- | --- | --- | --- | --- | --- | --- |
| D18-Cl:Y6 | 25.53 | 23.11 | 26.78 | 95.3 | 86.3 | 7.46×10^-4^ | 4.58×10^-4^ | 1.63 |
| D18-Cl:Y6:Y6-1O | 25.87 | 23.28 | 26.92 | 96.1 | 86.5 | 8.67×10^-4^ | 6.06×10^-4^ | 1.43 |
| D18-Cl: Y6-1O | 22.72 | 19.89 | 23.96 | 94.8 | 83.0 | 7.04×10^-4^ | 4.14×10^-4^ | 1.70 |

**Figure S4.** The *J-V* curves of the optimized D18-Cl:Y6 (a), D18-Cl:Y6:Y6-1O (b) and D18-Cl:Y6-1O (c) OPVs under different light illumination intensity, obtained from standard AM 1.5G (100 mW cm^-2^) illumination using a set of neutral optical filters.

**Table S3.** The key parameters of the optimized binary and ternary OPVs.

| Y6-1O content (wt%) | *R_1_* (Ω) | *R_2_* (Ω) | *C* (nF) | *R_3_* (Ω) | *CPE_P_* | *CPE_T_* (nF) |
| --- | --- | --- | --- | --- | --- | --- |
| 0 | 33.8 | 23.2 | 7.37 | 11.3 | 0.941 | 43.8 |
| 30 | 32.9 | 18.1 | 8.21 | 16.2 | 0.977 | 19.3 |
| 100 | 34.2 | 32.0 | 9.27 | 9.29 | 0.956 | 39.6 |

**Figure S5.** The plotted ln(*Jd*^3^/*V*^2^) versus (*V*/*d*)^0.5^ curves of typical binary and ternary OPVs.


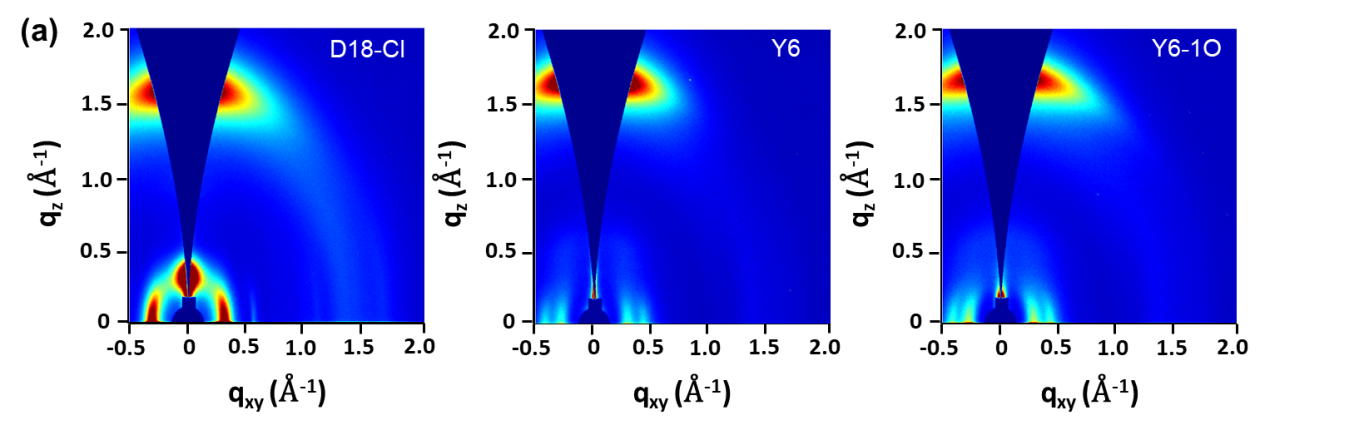

**Figure S6**. (a) The 2D-GIWAXS patterns of neat films. The out-of-plane (b) and in-plane (c) line-cut profiles of the GIWAXS patterns in neat films.

**Table S4**. The diffraction vector (*q*) values of diffraction peaks and crystal correlation length (*L_C_*) in neat and blend films.

| Neat/Blend films | Diffraction vector  (Å^-1^) | | | | Crystal correlation length (nm) |
| --- | --- | --- | --- | --- | --- |
|  | OOP  (100) | OOP (010) | IP  (100) | IP  (010) | IP  CCL_100_ |
| D18-Cl:Y6 | ─ | 1.70 | 0.31 | ─ | 5.3 |
| D18-Cl:Y6:Y6-1O | ─ | 1.71 | 0.31 | ─ | 5.8 |
| D18-Cl:Y6-1O | ─ | 1.72 | 0.31 | ─ | 5.1 |
| D18-Cl | 0.32 | 1.63 | 0.31 | ─ | 6.3 |
| Y6 | ─ | 1.71 | 0.28 | ─ | 4.5 |
| Y6-1O | ─ | 1.72 | 0.28 | ─ | 4.0 |
